# Efficacy of anifrolumab on skin, joint and lung involvement in anti-MDA5 positive dermatomyositis: a case report

**DOI:** 10.3389/fimmu.2025.1717766

**Published:** 2025-11-27

**Authors:** Federico Pettorossi, Laura Brischigliaro, Marisol Bracalenti, Aurora Di Gregorio, Beatrice Moccaldi, Roberto Depascale, Margherita Zen, Elisabetta Zanatta, Mariele Gatto, Andrea Doria, Luca Iaccarino

**Affiliations:** 1 Rheumatology Unit, Department of Medicine-DIMED, University of Padova, Padua, Italy; 2 Rheumatology Unit, Department of Clinical and Biological Sciences, University of Torino, Turin, Italy

**Keywords:** anti-MDA5, anifrolumab, ILD, skin ulcers, case report

## Abstract

A 55-year-old woman was diagnosed with anti-melanoma differentiation-associated gene 5 (anti-MDA5) positive dermatomyositis (DM) in 2020, having low grade fever, weight loss, arthritis in small joint of hands, erythematous-desquamative lesions on hands, cuticle dystrophy and severe skin ulcerations. Firstly, she was treated with cyclosporine (CsA), soon discontinued due to gastrointestinal intolerance. She was subsequently treated with steroid pulses, hydroxychloroquine (HCQ) and mycophenolate (MMF), without improvement. In March 2021 she started therapy with intravenous immunoglobulins (IVIG) and prostanoids, leading to ulcer improvement, but stopped due to gastrointestinal intolerance. A chest high resolution computed tomography (HRCT) done in June 2021 showed interstitial lung disease (ILD). In September 2021 rituximab (RTX) was stopped at the first infusion due to gastrointestinal intolerance. From January 2022 the patient also started to walk with difficulty due to development of deep asthenia. Therapy with various Jak inhibitors was started (first tofacitinib, then baricitinib, finally upadacitinib), leading to improvement of cutaneous ulcers, but stopped every time after a few months due to gastrointestinal intolerance and dizziness. In August 2023 during hospitalization a spirometry showed reduction of diffusion capacity of the lung for the carbon monoxide (DLCO). In June 2024, in consideration of poor disease control and refractoriness of the disease (loss of appetite and weight, worsening of asthenia which forced the patient into a wheelchair, persistence of polyarthritis, skin ulcers, alopecia, Gottron’s signs, radiological progression of ILD), she was hospitalized again and Anifrolumab (ANI) was started in July 2024 (300 mg IV every four weeks). After four infusions the patient reported improved appetite with significant weight gain, resolution of arthritis and disappearance of cutaneous ulcers, Gottron’s sign and alopecia. In February 2025, after seven ANI infusions, a HRCT demonstrated a significant radiological improvement of the ILD compared to 2024, and spirometry showed significant improvement of DLCO compared to 2023. In this period, no adverse effects were observed from the new therapy. After twelve total infusions, constitutional, articular and cutaneous involvement remained in good control. This case suggests the potential efficacy of ANI in refractory anti-MDA5-positive DM not only on skin manifestation, but also on articular and lung involvement.

## 1 Introduction

Idiopathic inflammatory myopathies (IIM) are a heterogeneous group of autoimmune diseases characterized by inflammatory involvement primarily of skeletal muscle. They can also affect many other organs such as skin, joints, lung and heart. DM is a subset of IIM typically characterized by muscle and skin inflammation (1). Myositis specific and associated autoantibodies can be detected in most of the patients affected by IIM, such as TIF1-γ, NXP-2, Mi-2, MDA5 and SAE, usually identifying a typical clinical phenotype (2).

Anti-MDA5 positive DM is a distinct clinical subtype of DM characterized by autoantibodies targeting MDA5, most often associated with hypo or amyopathic features, severe cutaneous manifestations including skin ulcers and a high risk of rapidly-progressive interstitial lung disease (RP-ILD) development (3, 4). The pathogenesis involves a combination of innate immune activation, autoimmunity and vasculopathy, but it is believed that the type I interferon (IFN) pathway plays a central role, as its signature has been shown to be upregulated in anti-MDA5 + DM (5). A triggering event, frequently a viral infection, occurs in a genetically predisposed individual leading to excessive MDA5 production, its mislocalization within cells, tissue injury and a loss of immune tolerance. Once anti-MDA5 antibodies are generated, they lead to inflammation and tissue damage, potentially initiating a cytokine storm (6). In this context, elevated IFN levels could contribute to blood vessel damage through toxic effects on the endothelium (7). The body’s attempt to repair the ongoing damage and cope with ischemia may then lead to the recruitment of macrophages, the development of fibrosis, and ultimately irreversible organ damage (8).

Anti-MDA5 DM treatment is individualized in consideration of the heterogeneity of clinical manifestations and their severity. Steroids typically are the first line of therapy, in refractory cases followed by immunomodulants or immunosuppressants such as hydroxychloroquine (HCQ), methotrexate (MTX), azathioprine (AZA), micophenolate mofetil (MMF), calcineurin inhibitors (CNIs), JAK inhibitors (JAK-i), intravenous immunoglobulin (IVIG) and rituximab (RTX) (9, 10). Most severe cases, especially due to RP-ILD, are treated with steroid pulses, plasma exchange therapy (PEX) and cyclophosphamide (CYC), eventually in association with CNIs (11, 12).

ANI is a human monoclonal antibody to type I IFN receptor subunit 1 currently used in patients with SLE, due to the interferogenic signature involved in its pathogenesis (13). Therefore, it is a promising therapy for interferogenic manifestations of anti-MDA5 + DM such as cutaneous, articular and pulmonary involvement. Specifically, it could be effective in treating the most severe manifestations of this subset of DM such as cutaneous ulcers, polyarthritis and ILD, maybe also the RP-ILD.

In this paper we describe a patient affected by anti-MDA5 + DM with cutaneous, articular and pulmonary involvement refractory or intolerant to multiple immunosuppressive therapies successfully treated with ANI.

## 2 Case description

A 55-year-old Caucasian female patient, non-smoker, with no past medical history nor familiarity for autoimmune and rheumatologic diseases, was diagnosed with anti-MDA5-positive DM in July 2020. The disease started in February 2020 with Gottron’s papules on hands and erythematous rash on the neck and arms, followed by hand arthralgias, fever (maximum body temperature of 38.2 °C) and progressive weight loss. At the time complete immunological panel was performed, showing negativity of anti-nuclear antibodies (ANA), anti-extractable nuclear antigens antibodies (anti-ENA), rheumatoid factor (RF), anti-citrullinated protein antibodies (ACPAs), and positivity of anti-MDA5 antibodies. The only relevant result in routine blood exams was a slight polyclonal hypergammaglobulinemia with a concomitant mild increase of serum immunoglobulin G (IgG). Complete blood count (CBC), inflammatory markers, muscle enzymes, transaminases, proteins, albumin, IgM, IgA, creatinine and urine exam were in normal range.

**Figure 1:**
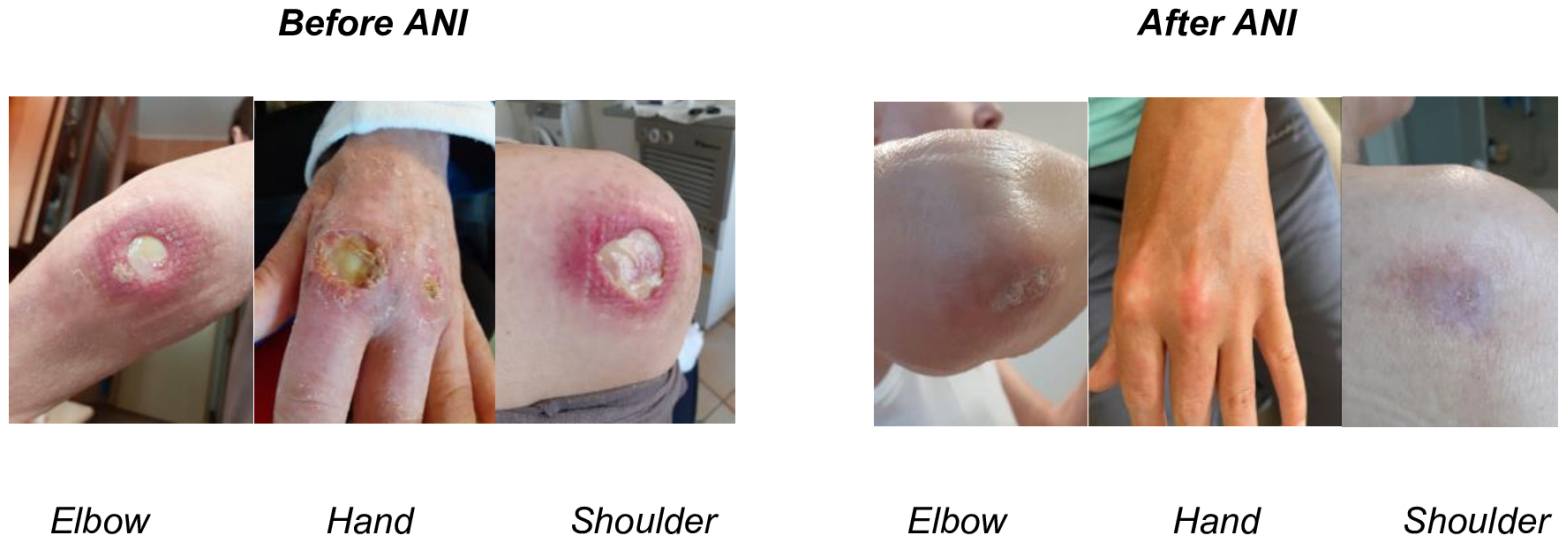
Cutaneous involvement before ANI (left side) and correspondent localizations after ANI (right side).

In July 2020 she started prednisone 50 mg daily (1 mg/kg/day) with mild improvement. In the same period a capillaroscopy was performed, showing a pattern of a specific microangiopathy. Suspecting an amyopathic DM, at the end of July 2020 she was hospitalized. During her hospitalization many examinations were performed. Joint ultrasound (US) confirmed metacarpophalangeal (MCP) and proximal interphalangeal (PIP) arthritis. Chest X-ray, mammography and abdomen US showed no significant abnormalities. On 4^th^ August 2020 she self-discharged, at that time she was taking prednisone 25 mg daily (0,5 mg/kg/day). In the same month she performed HRCT that showed no ILD, lymphadenopathies nor pleural effusion. In October 2020 she started therapy with CsA, discontinued shortly after due to gastrointestinal discomfort. In November 2020 she was treated with steroid pulses (methylprednisolone 250 mg daily for three days) without significant improvement on the cutaneous and articular manifestations. Furthermore, in the same period cutaneous ulcers appeared on hands, elbows, shoulders and heels (**Figure 1**), therefore in December 2020 she started therapy with MMF 3 g daily and HCQ 300 mg daily. As the patient developed oral ulcers, MMF was temporarily reduced to 2 g daily. Since March 2021, in consideration of persistent cutaneous and articular manifestations, monthly IVIG dosed at 0,4 mg/kg and prostanoids infusions were added, leading to ulcers mild improvement. Unfortunately, both IVIG and prostanoids were stopped in June 2021 due to post-infusion headache and gastrointestinal intolerance. Concomitantly, a new HRCT showed the development of mild ILD. In September 2021 she was treated with RTX, but the first infusion was prematurely stopped due to malaise and gastrointestinal intolerance. Therefore, in January 2022 she started therapy with JAK-inhibitor (tofacitinib) that led to significant ulcers improvement; this drug was stopped in August 2022 for hypotension and gastrointestinal intolerance. In consideration of the good response to JAK-inhibitors, baricitinib was started, with stabilization of cutaneous manifestation, however nausea, headache and vertigo occurred and the drug was stopped after few weeks with regression of the symptoms. Due to recurrent gastrointestinal disorders, onset of loss of appetite with progressive weight loss and persistence of polyarthritis, in August 2023 the patient was admitted to our Rheumatology department. To evaluate her gastrointestinal symptoms, she carried out numerous investigations such as an abdominal ultrasound, an abdomen CT scan, an EGDS and an MR cholangiography, which ruled out possible organic causes. She also performed a gastroenterological evaluation, which confirmed attribution of the symptoms to the medications the patient was taking at the time, as no other causes were identified. During hospitalization, hand polyarthritis was confirmed by clinical and US evaluation. She also performed spirometry that showed severe DLCO reduction (27%).

Subsequently, she was treated with steroid therapy (oral prednisone 10 mg/day) with transient benefit on articular and constitutional symptoms and in October 2023 another JAK-inhibitor was started (upadacitinib), without significant improvement of cutaneous ulcers and arthritis and with persistence of gastrointestinal discomfort. Therefore, the drug was stopped after few months.

**Figure 2:**
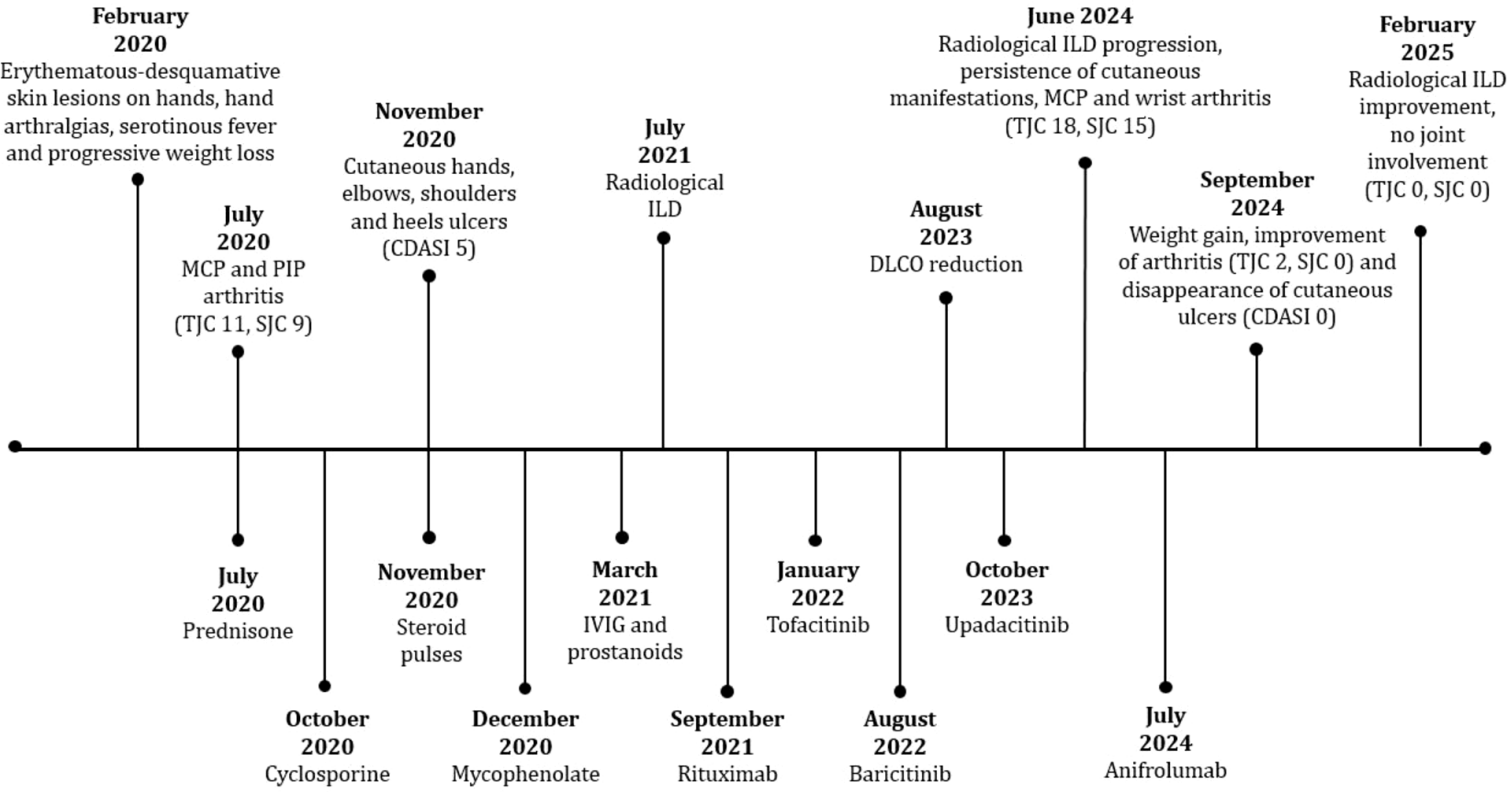
Timeline showing manifestation of the disease and corresponding treatments from diagnosis to starting of therapy.

In June 2024, in consideration of poor disease control and refractoriness (loss of appetite and weight, deep asthenia with forced ambulation in a wheelchair, persistence of hand polyarthritis with swollen joint count -TJC- of 18 and swollen joint count -SJC- of 15, skin ulcers and alopecia with cutaneous dermatomyositis disease area and severity index -CDASI- 5), she was hospitalized again. During her stay HRCT showed radiological progression of ILD. Therefore, she started therapy with ANI (300 mg IV every four weeks), firstly administered in July 2024 (**Figure 2**). At this time, she was taking 15 mg per day of prednisone, and the steroid decalage was started. No other disease modifying anti-rheumatic drugs (DMARDs) were prescribed concomitantly. After four infusions, the patient reported improvement of asthenia with recovery of walking (no more need of wheelchair), increased appetite with subsequent 6 kg weight gain (from 38 to 44 kg), marked improvement of arthritis (TJC: 2, SJC: 0) and disappearing of cutaneous manifestations, including ulcers (**Figure 1**) (CDASI 0). At this time, the patient was assuming 5 mg per day of prednisone, in further progressive decalage.

**Figure 3:**
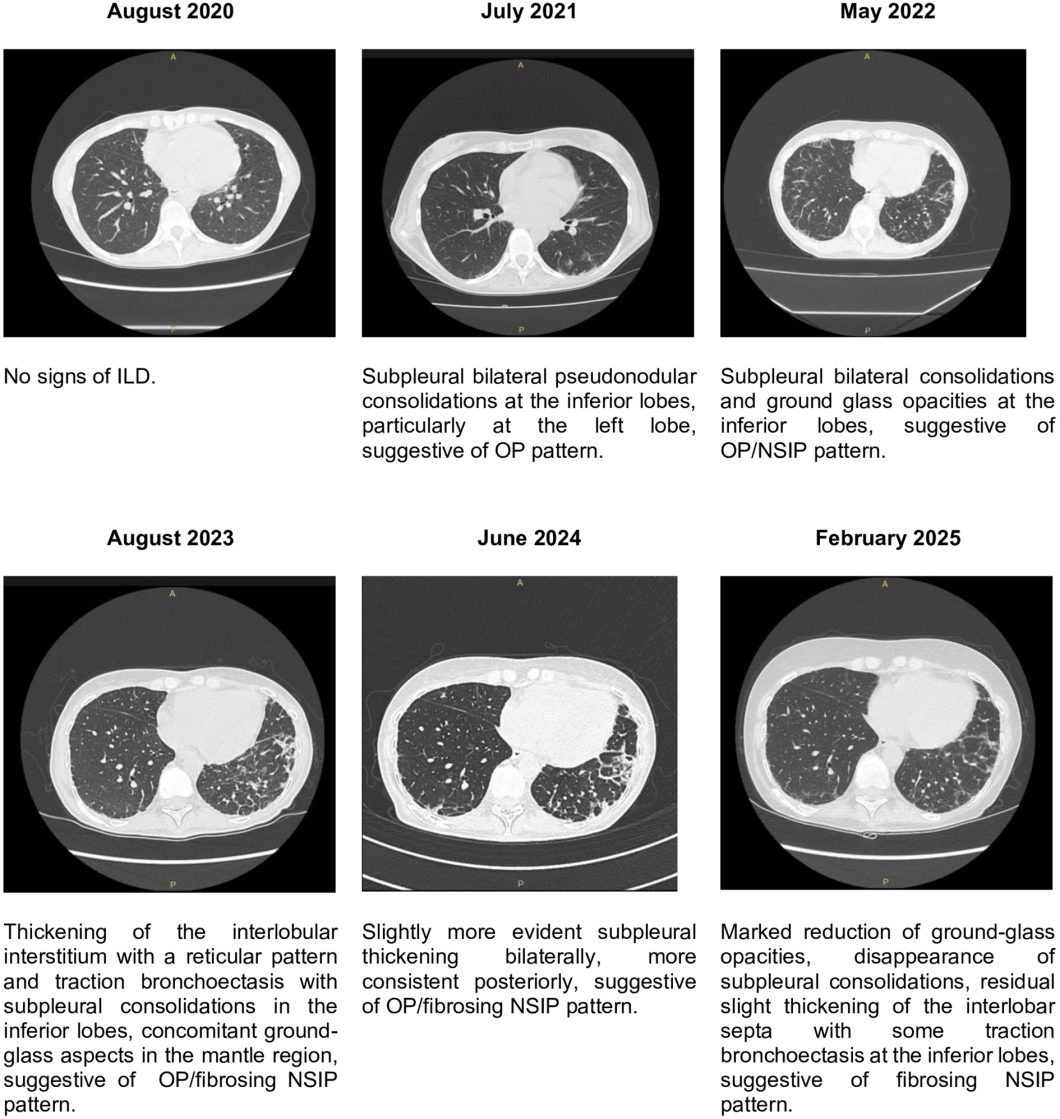
Images and radiological reports of lungs HRCT, showing the more important findings and the predominant ILD pattern.

Furthermore, in February 2025 the patient repeated HRCT and the case was discussed in a multidisciplinary medical team with dedicated pneumologists and radiologists, showing a significant improvement of the ILD compared to 2024 (**Figure 3**), and spirometry showing significant improvement of DLCO (47%) compared to 2023. In **Table 1** we summarized main clinical, radiological and functional data across different years of follow-up.

**Table 1:** clinical, radiological and functional data during follow-up, sorted by type of involvement.

Year	Skin	Muscular	Articular	Lung
July 2020	Gottron’s papules on hands; erythematous rash on neck and arms; cutaneous ulcers on hands, elbows, shoulders and heels.	Absent (normal CPK and MMT-8 150/150)	MCP and PIP polyarthritis (TJC: 11; SJC: 9)	Spirometry: not performed HRCT: no signs of ILD.
July 2021	Gottron’s papules on hands; cutaneous ulcers on hands, elbows and heels.	Absent (normal CPK and MMT-8 150/150)	No arthritis (TJC: 3; SJC: 0)	Spirometry: not performed HRCT: OP pattern.
May 2022	Cutaneous ulcers on hands and elbows; facial erythema.	Absent (normal CPK and MMT-8 150/150)	MCP polyarthritis (TJC: 9; SJC: 7)	Spirometry: not performed HRCT: OP/NSIP pattern.
August 2023	Cutaneous ulcers on hands.	Absent (normal CPK and MMT-8 150/150)	Wrists, MCP and PIP polyarthritis (TJC: 20; SJC: 17)	Spirometry: FEV1 73%, FVC 74%, TLC 74%, DLCO 27%. HRCT: OP/fibrosing NSIP pattern.
June 2024	Cutaneous ulcers on hands, elbows and shoulders; Gottron’s sign; alopecia.	Absent (normal CPK and MMT-8 150/150)	MCP and PIP polyarthritis (TJC: 18; SJC: 15)	Spirometry: FEV1 52%, FVC 57%, TLC 76%, DLCO 32% HRCT: OP/fibrosing NSIP pattern.
February 2025	Absent.	Absent (normal CPK and MMT-8 150/150)	No arthritis (TJC: 0, SJC: 0)	Spirometry: FEV1 86%, FVC 78%, TLC 76%, DLCO 47% HRCT: fibrosing NSIP pattern.

In this period of time, no adverse effects were observed from the new therapy. In July 2025, after a total of twelve ANI infusions, steroid was completely discontinued. In September 2025, after fourteen total infusions, constitutional, articular and cutaneous involvement persisted in good control, with absence of arthritis (TJC: 0, SJC: 0) and CDASI 0.

## 3 Diagnosis, therapy, follow-up, outcomes

From the diagnosis the patient was evaluated with regular ambulatory follow-up, six-monthly in periods of clinical stability and three-monthly in periods of poor disease control.

For each medical visit the patient repeated periodical laboratory exams, including complete blood count (CBC), C-reactive protein (CRP), creatine phospho-kinase (CPK), myoglobin, aldolase, lactate dehydrogenase (LAD), aspartate aminotransferase (AST), alanine aminotransferase (ALT), serum creatinine and urine exam.

Disease activity was assessed differently for each organ involvement.

Cutaneous involvement was assessed by clinical evaluation, using CDASI. After starting in July 2024, a significant cutaneous improvement was observed, as shown by CDASI reduction from 5 (Gottron’s sign, skin ulcerations and alopecia) to 0.

Articular involvement was evaluated clinically (TJC and SJC) and radiologically, with musculoskeletal US and conventional X-ray. After starting ANI, joint involvement improved as documented by complete disappearance of arthralgia and arthritis, consequently TJC decreased from 18 to 0 and SJC from 15 to 0.

Lung involvement was assessed by clinical evaluation, periodical global spirometry with DLCO and HRCT. Clinically she never experienced exertional dyspnoea neither dry cough nor other respiratory symptoms. In August 2020 HRCT showed no signs of ILD. In June 2021 HRCT showed initial ILD with organizing pneumonia (OP) pattern at inferior lobes. Subsequent HRCT performed in 2022, 2023 and 2024 showed slow ILD progression during years. In August 2023 spirometry showed mild reduction of lung volumes (FEV1 73%, FVC 74%, TLC 74%) and severe reduction of DLCO (27%). In March 2024 spirometry worsened with moderate volume reduction (FEV1 52%, FVC 57%, TLC 76%) and stability of DLCO (32%). In January 2025, after 6 infusions of ANI spirometry demonstrated stability of lung volumes (FEV1 86%, FVC 78%, TLC 76%) and significant improvement of DLCO (47%).

In February 2025 the last HRCT showed a significant ILD improvement compared to the previous one of 2024, confirmed by a multidisciplinary medical team with dedicated pneumologists and radiologists. We report that in August 2023, during the hospitalization in our ward, an echocardiogram was performed showing a concentric left ventricle remodeling and a mild reduction of ejection fraction (EF, 43%) in absence of altered chamber volumes, indirect signs of increased pressure in the pulmonary circulation (sPAP 23 mmHg) and pericardial effusion. No cardiac symptoms were reported at the time and cardiac enzymes (troponin I, NT-proBNP) were normal. A cardiologic evaluation suggested the execution of coronary CT and cardiac MRI, however the patient refused further investigations. In June 2024 another echocardiogram showed stability of EF (43%) and of the remaining echocardiographic findings.

Muscle involvement was evaluated clinically by performing manual muscle test 8 (MMT-8), that always resulted normal (150/150), and serologically with myocytolysis enzymes (CK, myoglobin, aldolase, LDH, AST, ALT), that always resulted in normal range. Moreover, the patient never reported muscle weakness.

## 4 Discussion and literature review

Based on our research, this is the first case report showing efficacy of ANI not only in skin manifestations, but also in lung and articular involvement in IIMs. Furthermore, to the best of our knowledge there are no data on the efficacy of in anti-MDA5+ DM.

Some case reports (14–19) have been published about the effectiveness of ANI in treating refractory cutaneous and muscular involvement of DM, as summarized in **Table 2**.

**Table 2:** Case reports on the efficacy of ANI in cutaneous and muscular involvement of DM.

Ref. No.	Authors (year) and study type	Associated antibody	Patient and diagnosis	Clinical presentation	Anifrolumab dosage	Follow-up duration	Outcome
(14)	Ang et al. (2023) - Case Report	Not specified	43-year-old woman with DM	Pruritic rash on face, hands, and torso	300 mg IV every 4 weeks	12 weeks	Complete resolution of heliotrope rash and Gottron’s papules within 12 weeks
(15)	Shaw et al. (2024) - Case Report	Anti-TIF1-γ	14-year-old girl with JDM	6-year history of pruritic rash on face, trunk, and extremities	300 mg IV every 4 weeks	8 weeks	Dramatic reduction in skin disease activity by week 4 of treatment
(16)	Shaw et al. (2024) - Multi-center retrospective cohort	Various (anti-TIF1-γ, anti-Mi-2, anti-NXP2)	Seven adults with DM	Pruritic photosensitive rash on face, neck, arms and hands	300 mg IV every 4 weeks	12 to 24 weeks	Majority of patients showed ≥50% improvement in CDASI scores by 3–6 months
(17)	Murthy et al. (2024) - Case Report	Anti-Mi-2	51-year-old woman with DM	Pruritic rash on face, neck, upper back and hands	300 mg IV every 4 weeks	12 weeks	Marked improvement in poikiloderma and scalp involvement; no relapse at follow-up
(18)	Chaudhary et al. (2025) - Case Report	Anti-NXP2	38-year-old man with DM	Photosensitive rash o face, upper trunk and thighs, muscle weakness	300 mg IV every 4 weeks	24 weeks	Sustained clearance of rash and no disease flare over follow-up period
(19)	Shayegan et al. (2025) - Case report	Not specified	16-year-old young man with JDM	Rash on face, upper back and chest and arms, proximal myositis	300 mg IV every 4 weeks	24 weeks	Improved skin symptoms, improved MMT and normalized muscle enzymes
(20)	Galluzzo et al. (2025) - Case report	Anti-TIF1-γ	34-year-old woman with DM	Rash on face and hands	300 mg IV every 4 weeks	8 weeks	Dramatic reduction of skin inflammation (CDASI from 34 to 4)
(21)	Helm et al. (2025) - Case report	Anti-TIF1-γ	66-year-old woman with DM	Rash on face, scalp, chest, shoulders and back	300 mg IV every 4 weeks	>52 weeks	Significant improvement in erythema and pruritus (CDASI from 26 to 11)
(22)	Claudio-Oliva et al.	Anti-TIF1-γ	53-year-old woman with DM	Rash on face, scalp, hands and trunk	300 mg IV every 4 weeks	12 weeks	Rapid reduction of skin activity and significant clinical improvement

Of these patients, three had juvenile DM (15, 19) while the others had adult-onset DM (16–18, 20); two patients had paraneoplastic DM (14, 22). Of the six cases that reported autoantibodies, four had anti-TIF1-γ autoantibody (15, 20–22), one had anti-Mi2 autoantibody (17) and one had anti-NXP2 autoantibody (18), while none of them had anti-MDA5-positive autoantibodies. All patients were treated with 300 mg of ANI intravenously every four weeks, and none of them reported adverse effects related to ANI during the follow-up period.

All case reports described the efficacy of ANI in DM as mainly limited to cutaneous manifestations with significant reduction of CDASI, when reported, only one case also described improvement in muscle involvement (19). In contrast, in our anti-MDA5–positive DM patient ANI demonstrated broader efficacy, significantly reducing arthritis and interstitial lung disease and improving skin disease not only in classical erythematous manifestations but also in ulcers, thus extending the spectrum of observed therapeutic benefits. Notably, in our patient marked improvement of refractory ulcers, polyarthritis and lung involvement was achieved soon after starting of ANI and in the same period no other therapeutic changes were made and the patient was in treatment only with ANI. For these reasons, it is reasonable to conclude that these improvements were achieved by ANI. Limitations arise from the nature of case report, which shows a single clinical case. To demonstrate the efficacy of anifrolumab in IIMs, we are awaiting the outcome of the ongoing randomized controlled phase III trial (23).

## 5 Patient perspective

After ANI initiation the patient’s quality of life significantly improved, regaining autonomy in walking and leaving the wheelchair. It has already been 14 months since the start of treatment and she is satisfied since until now it was the only treatment both effective and well tolerated among her medical history.

## Data Availability

The raw data supporting the conclusions of this article will be made available by the authors, without undue reservation.
